# ID 38 - Desenvolvimento de Ferramenta para Apoio ao Planejamento Estratégico de Ações em Avaliação de Tecnologias em Saúde em Secretarias Estaduais de Saúde

**DOI:** 10.5327/2237-9622.2025.v34s1.38

**Published:** 2025-11-25

**Authors:** Barbara Cristiane da Silva, Ana Paula Blankenheim, Bruna Marmett, Rodrigo Pereira de Almeida, Marina Petrasi Guahnon, Muriel Primon de Barros, Roseana Boek Carvalho, Kelli Carneiro e Freitas Nakata, Jéssica Escorel Chaves Cavalcanti, Rafhaella Cedro, Gilson Pires Dorneles, Maicon Falavigna, Suena Medeiros Parahiba

**Keywords:** avaliação de tecnologias em saúde, planejamento estratégico, Secretarias Estaduais de Saúde

## Abstract

**Introdução:**

O desenvolvimento e o fortalecimento de avaliações de tecnologias em saúde (ATS) em Secretarias Estaduais de Saúde (SES) possuem potencial para melhorar a eficácia operacional, fortalecendo as estruturas locais por meio do apoio à tomada de decisão no contexto estadual, além da promoção da colaboração entre os estados. Por outro lado, as práticas e as ações em ATS ainda estão na fase inicial no contexto estadual, necessitando aprimoramento em aspectos como regulamentação e aperfeiçoamento de atividades técnico-profissionais. Esta ferramenta tem como objetivo contribuir no processo do planejamento estratégico, e assim apoiar no desenvolvimento de ações de ATS no âmbito das SES.

**Método:**

A elaboração do planejamento estratégico para ATS integra as atividades do projeto ATS Educação, uma iniciativa entre o Hospital Moinhos de Vento, o Conselho Nacional de Secretarias de Saúde e o Ministério da Saúde, para disseminar e aperfeiçoar a ATS nas SES. O início da atividade ocorreu após a identificação da estrutura e os processos relacionados a ATS, por meio de um diagnóstico situacional, nas SES de 26 unidades federativas do Brasil. Para a organização do planejamento, as SES foram agrupadas com base nas características identificadas no diagnóstico situacional, considerando aspectos como infraestrutura, produção, recursos financeiros e humanos para ATS e priorização da gestão, e o instrumento com indicadores de planejamento estratégico foi elaborado para cada grupo. Para apoiar o desenvolvimento do planejamento junto às SES, um instrumento de planejamento de ações foi construído utilizando o Google Planilhas, tendo como base os materiais disponibilizados pela Rede Brasileira de Avaliação Tecnologia e Saúde (Rebrats) que orientam a implementação de núcleos de ATS (Nats). De forma resumida, o instrumento de planejamento é constituído por atividades e metas com vistas à formação de recursos humanos, formalização e ampliação de estruturas de ATS nas SES em médio e longo prazo. Uma oficina com especialistas em ATS estadual foi realizada por meio de uma videoconferência para validar o instrumento.

**Resultados:**

Foram definidas três estratégias principais para o planejamento estratégico: 1) a elaboração de produtos de ATS conforme as diretrizes metodológicas; 2) o apoio e o fortalecimento das ações de ATS nas SES e a estruturação dos Nats das SES, e; 3) o monitoramento e a avaliação dessas ações. Inicialmente, foram identificados 14 objetivos e 22 indicadores, dos quais 15 são quantitativos absolutos e 7 dicotômicos. Após a oficina, foi incluído um indicador, totalizando 23 indicadores, para apresentação quantitativa, bem como a alteração na redação de 4 indicadores e 1 meta.

**Conclusão:**

A análise e os ajustes no instrumento destacam a importância da colaboração para elaborar ferramentas estratégicas que atendam melhor às necessidades das SES. Um planejamento estratégico conduzido de forma conjunta com os principais envolvidos no processo facilita a cooperação para fortalecer as atividades de ATS em contextos específicos, como nas SES. Futuras perspectivas englobam a personalização dos indicadores para cada SES, conforme a estrutura de ATS mapeada e a definição individual das ações com os profissionais de ATS das 26 SES

